# Genetic and non‐genetic drug resistance: Darwin or Lamarck?

**DOI:** 10.1002/1878-0261.13601

**Published:** 2024-02-02

**Authors:** Mariangela Russo

**Affiliations:** ^1^ Department of Oncology, Molecular Biotechnology Center University of Torino Italy

**Keywords:** drug resistance, epigenetic, genetic, tumor evolution

## Abstract

Drug resistance represents a major limitation to the long‐term efficacy of anti‐cancer treatments. The commonly accepted view is that the selection of inheritable genetic mechanisms governs the development of secondary resistance. However, compelling evidence suggests an important role for adaptive cell plasticity and non‐genetic mechanisms in the development of therapy resistance. The two phenomena are not mutually exclusive and the interplay between genetic and non‐genetic mechanisms may affect tumor evolution during treatment. A broader characterization of the genetic and non‐genetic mechanisms of drug resistance may pave the way for more precise and effective therapeutic strategies to overcome resistance.

## Graphical abstract

Genetic and non‐genetic mechanisms of drug resistance may co‐occur and co‐operate in cancer cell populations providing fitter cellular states able to resist to the lethal effect of anti‐cancer therapies. Notably, both Darwinian selection and Lamarckian drug‐induced adaptation may be involved in the development of drug resistance. A broader characterization of these phenomena is warranted to guarantee more effective therapeutic approaches.

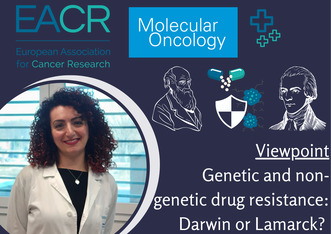

**Abbreviations**
DTPs: drug‐tolerant persisters
ITH: intra tumor heterogeneity

Despite the promising advances in cancer care and the improved knowledge of the biology and genetics of tumors, the eradication of the disease remains an unmet clinical challenge, with development of resistance as a major obstacle to overcome.

Cancer is a complex disease, characterized by a high level of genetic instability and intratumor heterogeneity. The first to theorize the process of tumor evolution was Nowell in 1976 [1]. According to Nowell's model, cancer originates and evolves from a single normal cell through a stepwise accumulation of genetic alterations conferring growth advantage. Selective pressures throughout cancer development drive sequential waves of clonal expansions and shape tumorigenesis. This model is in line with Darwin's theory of natural selection and survival of the fittest individuals, thus suggesting a linear tumor evolution. However, the advent of high‐depth next‐generation sequencing technologies unveiled a much more complex situation. Tumors are indeed highly heterogeneous and heterogeneity might already be present in the early stages of tumor development, with the simultaneous coexistence of multiple co‐dominant sub‐clones, therefore suggesting a branched, rather than a linear, tumor evolution [2, 3].

Tumor heterogeneity poses a serious impediment to tumor eradication and prevention of disease recurrence. It is widely held that cells harboring genetic mechanisms of resistance might already be present in the tumor lesion, albeit at low frequency, before treatment administration (Fig. 1). Therefore, the time to recurrence is the time necessary for pre‐existing mutant cells to repopulate the neoplastic lesion [4]. In this framework, while therapies eradicate sensitive cells, at the same time a constant and sustained treatment alters the tumor microenvironment, thus selecting pre‐existing resistant subclones that become fitter and outgrow other clones [5].

**Fig. 1:**
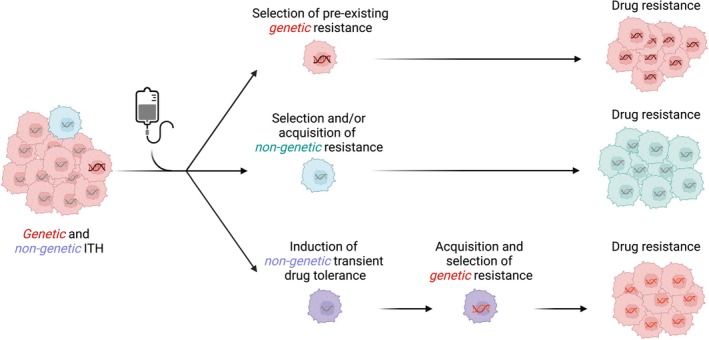
Genetic and non‐genetic mechanisms drive resistance to anti‐cancer therapy. Tumors may display genetic and/or non‐genetic intra tumor heterogeneity (ITH). Genetically drug‐resistant cancer cells, responsible for tumor recurrence, may be present in the tumor lesion before drug administration (upper panel). Pre‐existing (middle panel) or drug‐induced (lower panel) non‐genetic phenotype(s) may provide a fitter cellular state capable of tolerating drug exposure and drive drug resistance. Adaptive non‐genetic phenotype may favor the acquisition of genetic mechanism(s) of drug resistance (lower panel). Figure was created with BioRender.com.

Under this prism, some questions inevitably arise. Is cancer treatment doomed to fail before therapy administration? Does the treatment accelerate the emergence of more aggressive resistant clones? According to this, would it be better to maintain a stable population of drug‐sensitive cells thus limiting outgrowth of resistant ones?

In a substantial fraction of patients, anti‐cancer therapy leads to long‐lasting tumor shrinkage and clinical response, with relapse occurring only after prolonged disease stabilization [5, 6]. In this instance, the emergence of drug resistance cannot simply be explained by pre‐existing inheritable genetic alterations and suggests that drug resistance might go beyond Darwinian selection of resistance.

Emerging findings confirm that non‐genetic mechanisms might be equally important in shaping the evolution and adaptation to the drug‐induced hostile environment, as drivers of drug tolerance or facilitating the acquisition of genetic resistance [7, 8] (Fig. 1). Non‐genetic alterations such as chromatin remodeling, epigenetic modifications, transcriptional rewiring, and metabolic reprogramming are thought to promote intrinsic diversity and phenotypic plasticity within a population of cells. In agreement with this, phenotypically distinct but genetically identical cells could display different fitness and different ability to cope with a drug‐induced altered microenvironment (Fig. 1).

When cancer cells are challenged with therapy, the emergence of a reversible drug‐tolerant persister phenotype has been identified across a wide range of tumors in response to different anti‐cancer agents [9, 10]. Drug‐tolerant persisters (DTPs), widely considered the major components of minimal residual disease, survive to lethal doses of drugs without genetic mechanisms of resistance and represent a reservoir from which genetically divergent mechanisms of resistance might eventually emerge [11], thus playing a critical role in the failure of anti‐cancer therapy.

Notably, if DTPs are released from drug pressure, they re‐gain sensitivity to the same therapeutic regimen, thus arguing in favor of a non‐genetically driven drug tolerance state. In line with this, in some instances, pharmacological interference with chromatin modulators or altered metabolism proved effective in overcoming drug refractoriness [10, 12].

Importantly, results from different studies suggest that tumors may also display significant non‐genetic heterogeneity, besides genetic heterogeneity [13]. Whether persistence derives from the Darwinian selection of pre‐existing subclones or whether it occurs through a Lamarckian induction upon exposure to stress (e.g. by anticancer therapy), is still an open debate in the field. It is also unclear whether all cancer cells within a population are able to switch to a persister state, or whether only a fraction of cells is somehow predisposed to switching.

We recently unveiled that targeted therapies induce the switch from sensitive to persister phenotype in colorectal cancer and that this phenotype is mainly (if not solely) drug‐induced. The sustained exposure to drug‐induced stress further induces a temporary increase of the mutation rate of surviving persister cells, thus increasing the probability that alterations conferring resistance might be acquired [8, 14].

These results suggest that cancer cell plasticity and adaptive changes occurring as an active response to anticancer therapy can be considered as a form of Lamarckian adaptation, at least in colorectal cancer.

Darwinian selection and Lamarckian adaptation are not mutually exclusive but may co‐occur in the same cancer population and operate together in a synergistic manner, with a pre‐existing or induced non‐genetic phenotype providing a cellular state capable of tolerating the initial exposure to the drug. A continuous exposure to the drug may then induce the acquisition of adaptive mechanisms that are further selected, thus establishing a permanent drug resistance (Fig. 1).

Growing evidence has showed that cancer patients who become refractory to anti‐cancer therapy can be re‐challenged with the same therapeutic regimen after a period of drug holiday [5, 15]. This can have a double interpretation: (a) intermitting dosing allows the maintenance of a stable drug‐sensitive population thus limiting the expansion of pre‐existing, and often more aggressive, resistant cells, or (b) the release from drug pressure may preclude the complete adaptation of cancer cells to the stressful environment, thus favoring the reversion to the drug‐sensitive phenotype.

In our efforts to prevent or overcome drug resistance, we have significantly improved our knowledge on the identification and characterization of genetic drug resistance, but we have probably overlooked the non‐genetic/epigenetic role in drug resistance. The concept that resistance is *fait accompli* has a significant impact on the clinical treatment of cancer patients, as it implies that relapse will inevitably occur, leaving us hopeless.

Importantly, considering that tumors may be frequently exposed to harsh or changing tumor microenvironments, non‐genetic reversible mechanisms can become a plastic source of cellular states capable to adapt and resist to transient hostile environmental conditions that cancer cells may encounter during tumorigenesis, metastatic dissemination, and drug exposure.

But, when and how does non‐genetic resistance occur? Does a cell need to undergo epigenetic changes to become genetically resistant? Does the reversible nature of non‐genetic mechanisms of adaptation and resistance offer novel and promising therapeutic options?

The integration of multiple approaches, for example combining epigenetic modifiers and/or metabolic inhibitors with chemotherapy or targeted therapies, or implementing adaptive therapeutic strategies using sequential or intermitting drug administration may possibly better interfere with the ability of cancer cells to switch phenotypes and acquire a drug‐resistant state.

In conclusion, a careful assessment of non‐genetic mechanisms and phenotypic diversity in drug resistance and a broader understanding of how they interact with genetic alterations or promote evolvability is needed. This might help in guiding treatment decisions which are currently based mainly on genetic biomarkers, thus enhancing our possibilities to fight drug resistance and prevent cancer recurrence.

## Conflict of interest

The author declares no conflict of interest.
